# Resonance modulated amplified emission from CdSSe nanoribbons

**DOI:** 10.1038/srep15071

**Published:** 2015-10-16

**Authors:** T. Wood, K. T. Cheung, Y. Foo, Y. K. Liu, J. A. Zapien

**Affiliations:** 1 Department of Physics and Materials Science and Centre of Super Diamond and Advanced Films (COSDAF), City University of Hong Kong, Hong Kong; 2 Yunnan Normal University, Kunming, China

## Abstract

We present evidence of amplified emission mediated by surface plasmon polaritons (SPPs) from a CdS_0.2_Se_0.8_ nanoribbon (NR) supported on a gold-coated silicon substrate. Room temperature amplified emission is observed from the nanoribbon above excitation irradiances ~25 W/cm^2^ when it is supported on the gold coated silicon substrate. The nanoribbon is shown to act as a resonator cavity, leading to amplification of discrete wavelengths in the emission spectrum. Evidence for the formation of SPP waves between the gold-coated substrate and the nanoribbon is shown, and the resulting wavenumber increase allows for the matching of theoretical resonance wavelengths with those observed experimentally.

The field of semiconductor nanolasers has attracted a great deal of interest in recent years and such devices present many advantages over traditional laser technologies, the most striking being the range of output wavelengths available via tuning of the precursor material composition which cannot be matched by conventional techniques due to the lack of high crystallographic quality lattice-matching substrates^1^,^2^,^3^,^4^. An interesting goal for nanolaser research is to develop architectures possessing sub-wavelength dimensions that output continuous wave (CW) emission at room temperature under electrical injection^5^,^6^,^7^. Such an achievement would open up a myriad of applications taking advantage of the reduction in scale compared with that historically associated with tunable laser sources.

A wide range of nanolaser geometries, often grown through simple methods such as the vapor-liquid-solid (VLS) technique, have been investigated thus far; from simple nanowires^8^,^9^ to more complex extrusions with triangular^10^ or hexagonal^11^ cross sections. The tight two-dimensional confinement offered by such shapes, coupled with the strong refractive index contrast typically observed between the semiconductor material and its surroundings, make these structures ideal waveguides. Numerous publications take note of this fact, and seek to characterize the modal content of light trapped within the structure or exploit their strongly directional and polarized emission^12^,^13^,^14^.

It has been shown^15^,^16^,^17^,^18^ that light propagating in systems composed of dielectric nanostructures in close proximity to metallic surfaces undergoes a wavelength compression – or wavenumber increase – effect due to the formation of surface plasmon polaritons (SPPs). Consequently, light can be confined inside structures that have dimensions lower than the diffraction limit, typically defined in the context of nanolasers as being smaller than half of the emitted wavelength^19^. More broadly, other works have shown that the analysis of light confined in optically excited nanostructures must take into account a modified frequency-wavenumber dispersion relation in order to account for the spectral content of emitted radiation. This modification can be incorporated into an adjusted value for the refractive index of the material in which light is propagating, known as the effective material index. It is widely suggested that the origin of these perturbations to the dispersion relation lies in the coupling between optical and other electrical phenomena, with two main mechanisms evoked other than SPP formation: photon-exciton coupling^20^,^21^ or plasmon-exciton polariton coupling^22^. Similar to the case of SPPs, these effects allow for a modification of the wavenumber of light inside nano-scale systems. Many different structures for confining SPPs have been proposed, with most revolving around the use of metal nanostructures such as nanowires^23^,^24^ to limit the spatial extent of SPP waves. An alternative design enjoying popularity in the literature uses a semiconducting nanostructure as a gain-media and resonant cavity on top of a metal-coated substrate separated by a dielectric layer to define a hybrid plasmonic-photonic cavity^6^,^15^,^18^,^25^,^26^,^27^,^28^. The dielectric spacer is used to avoid unwanted charge transfer and its thickness mediates the interaction and coupling strength between the photonic and plasmonic modes.

In this paper, we analyse CW amplified emission at room temperature from a CdSSe nanoribbon that has been mechanically transferred to a gold coated silicon substrate. Analysis of the observed resonances based solely on the measured physical dimensions of the NR and the material refractive index fails to explain the modulated light emission. A strong modification of the effective index of the nanoribbon material is demonstrated that allows for the matching of theoretical and measured resonant wavelengths. This modification is attributed to the formation of SPP waves at the surface of the gold substrate that are laterally confined by the extent of the nanoribbon, the latter serving as a gain medium to compensate for ohmic losses in these hybrid photonic-plasmonic cavities. The evanescent field of the SPP wave couples with the electric field in the dielectric nanoribbon, thereby imposing an increase in the wavenumber of light confined therein as well as light confinement enhancement that explains the observation of amplified emission at excitation power densities above ~25 W/cm^2^.

## Results

### Nanoribbon synthesis

The CdSSe nanoribbons investigated were grown by the vapour-liquid-solid (VLS) method in a tube furnace via the temperature gradient method (TGM) to control their stoichiometric composition. This growth method, previously reported by our group^1^,^2^, may be used to tune the composition of ternary and quaternary^29^ semiconductor alloys. In our case, the binary alloy precursors were CdS, for which laser ablation is necessary in order to induce vaporization, and CdSe which is thermally evaporated inside the deposition chamber. Silicon substrates coated with ~20 Å gold film were placed within the tube furnace at a strategic position within the installed temperature gradient so as to determine the composition of the ternary alloy formed, that is to say the value of *x* in the formula CdS_1-*x*_Se_*x*_ is determined by the substrate temperature and hence its position following the axial direction in the tube furnace. Additional details of the synthesis and characterization of the resulting structures can be found in^30^.

**Figure 1:**
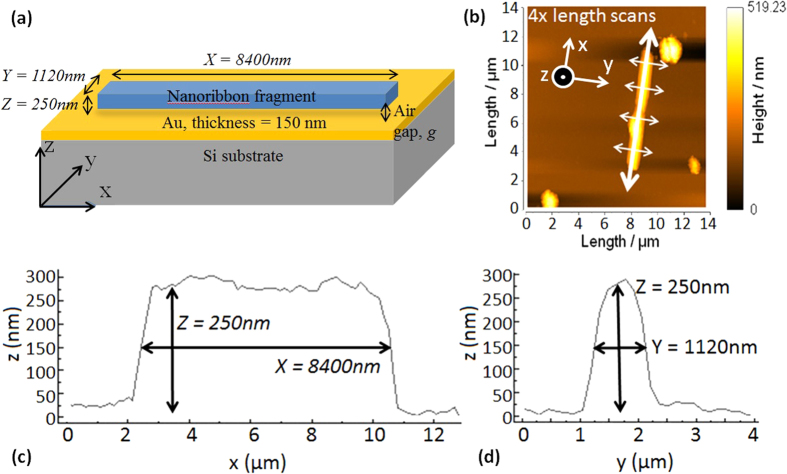
(**a**) Diagram showing nanowire on Au coated Si substrate with coordinate system and dimensions defined. (**b**) 14 × 14 μm surface scan image of selected nanoribbon showing width and length scan positions, dimensions calculated from (**c**) average of four length scans (**d**) average of four width scans.

Ternary CdS_1-*x*_Se_*x*_ nanoribbons (0 ≤ *x *≤ 1) were obtained from the VLS-TGM synthesis as previously reported^29^. After preparation, some nanoribbons were transferred to a silicon substrate coated with ~150 nm gold film prepared by D.C. magnetron sputtering (320 V) in a stainless steel chamber first evacuated to a base pressure ~2 × 10^−6^ mbar and backfilled with Ar to 2 × 10^−3^ mbar; the gold target (diameter ~7.5 cm) was ~10 cm away from the substrate. Close inspection by atomic force microscopy (AFM) revealed that the Au film was composed of islands with ~70 nm in-plane diameter. The morphology of the gold film, in particular its intrinsic roughness, is deemed to be instrumental in limiting losses due to exciton recombination, as will be discussed in more detail later. The resulting structure is that of Fig. 1 (a).

### Nanoribbon characterization

AFM measurements on both the nanoribbons and the gold substrates were performed with a Nanonics MV1000. Figure 1 (b) shows the AFM data in along with the longitudinal and transversal line scans (white arrows) used to estimate the average cross sections (Fig. 1 (c,d)) and to calculate the nanoribbon dimensions as 8400 nm in length (*x*), 1120 nm in width (*y*) and 250 nm in thickness (*z*).

The resonance enhanced PL spectra of the near band edge (NBE) emission of the nanoribbon for different excitation irradiances were collected with a Renishaw in-Via Raman spectrometer coupled to the AFM. All measurements were obtained at room temperature under continuous wave (CW) optical pumping using the 633nm line of a HeNe laser. The near band edge (NBE) emission of the single nanoribbon in Fig. 1b is ~680 nm corresponding to an estimated composition of *x*~0.8 following Vegard’s law^31^,^32^, which has been show to hold for self-assembled nanostructures^29^,^31^. Accordingly, its estimated refractive index is ~2.75 at 680 nm^33^.

**Figure 2:**
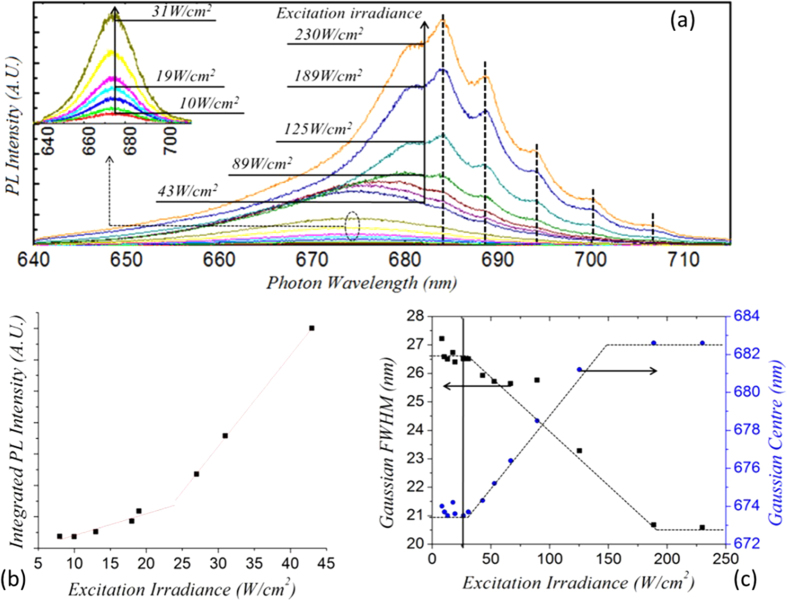
(**a**) Photoluminescence spectrum for selected nanoribbon as a function of the excitation irradiance (inset: excitation irradiances below 43 W/cm^2^ on expanded *y*-scale for clarity). (**b**) Integrated intensity as a function of excitation irradiance, showing onset of amplified emission around 25 W/cm^2^. (**c**) Evolution of FWHM and center position of Gaussian fitting curves of the PL spectra shown in (**a**) as a function of excitation irradiance. The lines are a guide to the eye.

The excitation beam was focused onto the sample at normal incidence using a 50 × microscope objective which was also used to collect the emitted light. The excitation irradiance was varied between 8 and 230 W/cm^2^ by adjusting the sample height with respect to the focal plane of the objective. Figure 2 (a) shows the collected PL emission from the NR supported on the gold-coated substrate for varying excitation irradiances. The onset of amplified emission is visible for irradiances higher than 25 W/cm^2^, as evidenced by the superlinear increase in the integrated intensity, shown in Fig. 2 (b). It should be noted that the integrated intensity is only shown for excitation irradiances <50 W/cm^2^ since at higher values the excitation beam diameter becomes smaller than the nanoribbon dimensions resulting in non-homogeneous illumination and the integrated intensity is expected to fluctuate strongly^17^. Figure 2 (c) shows the evolution of the full width half maximum (FWHM) and the centre position of a Gaussian fitting applied to the PL spectra as a function of the excitation irradiance. A narrowing of the FWHM and a red-shift of around 9 nm of the centre position is observed above 25 W/cm^2^, further confirming the onset of amplified emission. The observed shift is due to changes in the dominant light emission mechanism when transiting from spontaneous to amplified emission. During spontaneous emission, below 25 W/cm^2^ in Fig. 2 (a), the PL is dominated by NBE emission resulting from multiple processes and with center wavelength ~673 nm. However, as the excitation irradiance increases the amplified emission is dominated by the particular mechanism responsible for gain in a given nanostructure. In ZnO it has been suggested that excition-exciton scattering results in increased recombination^12^ while in CdS, it has been shown that phonon-scattering dominates at temperatures >75 K^13^. In fact, the LO phonon energy in CdS is ~38 meV and is equivalent to a red shift of ~7.6 nm which corresponds well with the observed ~9 nm shift when considering the composition (CdS_0.2_Se_0.8_) of the investigated nanoribbon.

In addition, for excitation irradiances above 30 W/cm^2^, resonant peaks are seen at wavelengths above 680 nm that are attributed to the formation of cavity modes within the nanoribbon as the gain overcomes losses. The left-most column of Table 1 groups these resonant wavelengths.

### Analytical modelling of resonant cavity modes

The classical treatment of the resonance phenomena is not sufficient to model the resonant effect observed in Fig. 2 (a). The free space resonant wavelengths *λ*_*0,lm*_ are often found using the following well-known equation for rectangular cavity resonances (Eq. 1), wherein *n* represents the material refractive index (estimated to be ~2.75 from Ref. 33), *l* and *m* are the longitudinal and transverse modal orders representing the number of field nodes in the x and y directions respectively, and *X* and *Y* represent the cavity length and width (i.e. the NR dimensions) respectively.


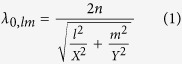


Upon inserting *m* = 8, for reasons that are explained later, and varying *l* in order to minimize the difference (as quantified by the chi squared estimator, *χ*^2^, see Eq. 7 below) between the experimental and theoretically obtained resonance wavelengths, one cannot obtain a satisfactory agreement between the latter, as shown in Table 1. It will be shown that a strong modification of the effective refractive index of the NR must be taken into account in order to successfully predict the experimentally observed resonance wavelengths.

One approach taken to explain anomalous material effective index dispersion in optically pumped media involves photon-exciton coupling phenomena, particularly when such dispersion occurs for photon energies near the band gap energy. It has been shown^17^,^18^ that tuning the oscillator strengths in the polariton equation that describes the dispersion induced by such coupling can match theoretical and observed resonant wavelengths in dielectric nanostructure cavities. In this case, higher excitation irradiances lead to higher exciton concentrations in the dielectric and hence the material effective index is modified as a function of the pump power, leading to a displacement of the resonant wavelengths observed. In our study the resonant wavelengths remain unchanged over the range of excitation irradiances used, as shown in Fig. 2 (a), and as such photon-exciton coupling is not thought to play an important role.

Further examination of the literature reveals that similar systems to the one under study have been documented previously, consisting of SiO_2_ dielectric stripes applied by electron beam lithography directly onto the surface of gold films to form waveguides^34^,^35^. In this case, 1D confinement in the plane of the gold substrate is supplied by the dielectric stripe and it is demonstrated that the measured modal index of propagating waves is that of confined SPP waves, as expected at a metal-dielectric interface. We therefore extend this confinement regime to the 2D situation with our nanoribbon upon a gold substrate, with the roughness of the substrate making the situation slightly more complex by introducing an air-gap between the metal and the dielectric.

Following the approach proposed by Ma *et al.*^6^, the analysis of light propagation in the structure may be decomposed so as to analyze the SPP behaviour and the cavity modes separately. In a slight modification to this method, a 2D *yz*—cross section of the nanoribbon is analysed to find the effective index of plasmonic modes assuming an infinite length along the nanoribbon’s axis (*x*- direction). A second 2D geometry in the plane of the ribbon (*xy*—section) is then used to find the cavity modes via a k-space analysis method^36^, converting the effective index from the plasmonic mode to an effective index of the nanoribbon material.

**Figure 3:**
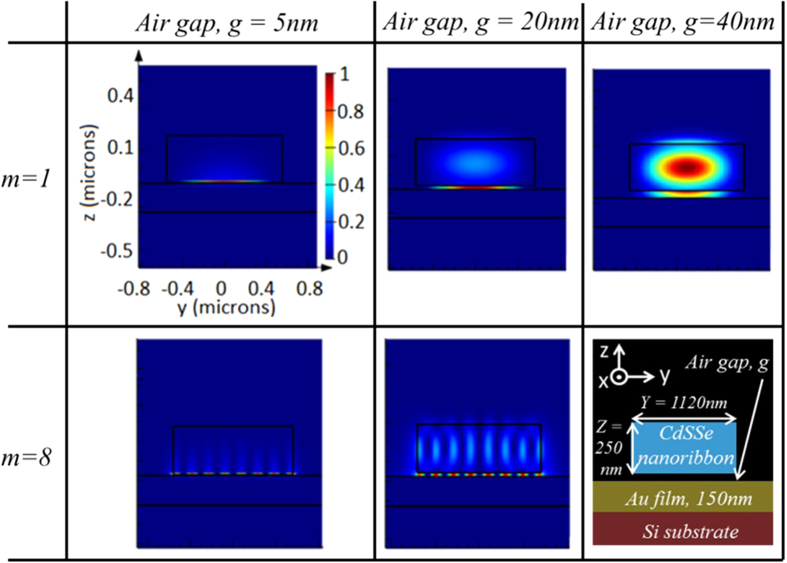
Electric field intensity ( = *E*_*x*_^2^* + E*_*z*_^2^) distributions for modal orders *m* = 1 and *m* = 8 for various air gap thicknesses (note that *m* = 8, *g* = 40 nm corresponds to a leaky mode and is not included). The scales shown on the plot [*g* = 5 nm, *m* = 1] apply to all others. Geometry for eigenmode solver simulations is a cross section of the structure in the *yz* plane.

The 2D *yz* cross section is shown in Fig. 3, along with SPP modal electric field profiles that correspond to the associated modal effective indices *N*_*mode*_ and losses *L*_*mode*_ found at λ_*0*_ = 680 nm using a commercially available eigenmode solver (Lumerical Mode Solutions) for selected values of *m* and air gap thickness *g*. The eigenmode solver takes as inputs the spatial configuration of the waveguide in the *yz* plane, which is assumed to be of infinite length perpendicular to this plane, as well as the complex refractive index of all the component materials. The results of the calculation for each guided mode are a map of the electric field in the structure and the complex modal wavevector^37^, *k = k*_*0*_. *(N*_*mode*_* + i.L*_*mode*_) where *k*_*0*_ is the free space wavenumber. The eigenmode solver returned 8 transverse magnetic (TM) modes for the structure over the full range of air gap thicknesses tested, which are numbered from *m* = 1 to *m* = 8, where *m* is the transverse modal order equal to the number of field nodes in the *y* direction. It should be noted that the modal effective indices for higher order modes (*m* ≥ 9) would decrease rapidly below 1 upon increasing the air gap thickness, and would therefore be leaky. All light confined in the modes is TM polarized, and is therefore susceptible to induce plasmonic phenomena at the gold film surface. The field profile for selected modes and air layer thicknesses is shown in Fig. 3.

**Figure 4:**
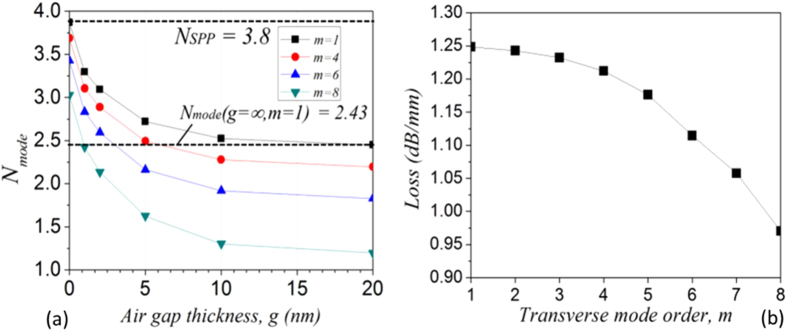
(**a**) Modal effective indices for selected modes as a function of air gap thickness. Limiting values for mode *m* = 1 are also shown. (**b**) Loss following the wavevector as a function of mode order for 5 nm air gap thickness.

A plot of the modal effective index as a function of the air gap thickness *g* for selected mode orders and a plot of the loss calculated by the mode solver (*L*_*mode*_ as before) for all modes for *g* = 5 nm are shown in Fig. 4. Clearly, as the modal order increases one can observe a decrease in the loss with increasing modal order as will be discussed below. This seems to agree with an apparent migration of the electric field intensity away from the loss-inducing gold layer into the core of the dielectric nanoribbon, as has previously been suggested^38^. When varying the size of the air gap *g* between the gold film and the nanoribbon, one observes for all modal orders that for small values of *g* the field is concentrated close to the metal surface in the air gap and the SPP modal characteristics of high loss and effective index dominate, whilst for larger *g* the field begins to concentrate in the dielectric.

As expected it can be seen that, for the fundamental mode (*m* = 1), as *g* tends towards zero the modal effective index tends towards the SPP effective index, given by^39^:


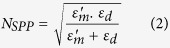


In Eq. 2, *ε*^*'*^_*m*_ is the real part of the dielectric constant of the metal and *ε*_*d*_ that of the dielectric. At 680 nm, these values are −16.1 and 7.6 respectively, yielding a value for the SPP effective index of 3.8. When *g* is over a few tens of nanometers, the modal effective index relaxes to that of a dielectric waveguide in free space, as verified for the mode *m* = 1 (*N*_*g*_ _*=*_ _*∞,m*_ _*=*_ _*1*_ = 2.43) with a standard mode solving algorithm for layered film stacks^40^.

Having obtained the dispersion of the effective index of the plasmonic mode as a function of the air gap thickness *g*, we seek to determine the nature of the modes giving rise to the amplified emission peaks seen in the experimental photoluminescence spectra (Fig. 2 (a) and left-most column of Table 1). To do this, we use the so called k-space analysis method in the *xy* plane, whereby the propagation vector is decomposed into orthogonal components following the length and width of the ribbon and related to its dimensions by Eq. 3 and Eq. 4. It can be shown that the free space wavelengths *λ*_*0,lm*_ undergo constructive interference upon round trips in the nanoribbon cavity that satisfy Eq. 5^41^.


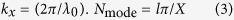



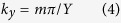



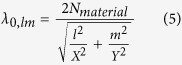


In Eqs. 3, 4, 5, *N*_*material*_ is the material effective index, *l* and *m* are the longitudinal and transverse modal orders representing the number of field nodes in the *x* and *y* directions respectively, and *X* and *Y* are the nanoribbon length and width respectively.

As illustrated in Fig. 4 (b), higher transverse modal orders correspond to lower loss due to greater penetration of fields into the dielectric; we therefore assume that *m* = 8 for all modes observed since we are interested on the resonant modes appearing after the onset of amplified emission where optical gain overcomes losses. Rearrangement of Eq. 3 allows us to determine the value of *l* at 680 nm: *l*_*680*_* = 2X.N*_*mode*_*/680* (for *X* in nanometers) and rearrangement of Eq. 5 and the substitution of all known values allows for the calculation of the material effective index, *N*_*material*_ at 680 nm, as a function of the air gap thickness *g*:


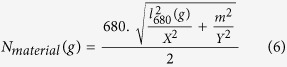


**Figure 5:**
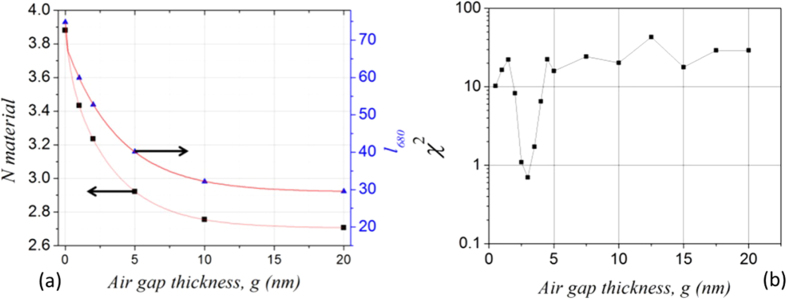
(**a**) Material effective index and longitudinal mode order at 680 nm (*l*_*680*_) as a function of air gap thickness. (**b**) Chi squared function applied to measured and theoretical resonance wavelengths as a function of air gap thickness, showing the true gap size to be around 3 nm.

This material effective index accommodates the wavelength compression phenomenon due to the presence of SPPs at the neighbouring gold surface. Plots of *l*_*680*_ and *N*_*material*_ as a function of *g* are shown in Fig. 5 (a). Taking into account the narrow spectral range of the photoluminescence observed, we assume that the dispersion of the material effective index is negligible and as such the values obtained at 680 nm are applied over the full range of wavelengths considered (680–710 nm). Finally, Eq 5 is evaluated as a function of *g* for the five values of *l* preceding the rounded-down integer value of *l*_*680*_, to yield a set of wavelengths that are compared to those measured experimentally. The final values for *N*_*material*_*, l*_*i*_ and *g* are those permitting the best agreement with the experimental data, as quantified by the chi squared parameter χ^2^ applied to the difference between the five measured *λ*_*exp*_ and theoretically obtained *λ*_*theory*_ resonance wavelengths:


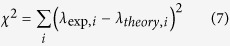


In our case these values are *N*_*material*_ = 3.10, 43 ≤ *l* ≤ 47, and *g* = 3.1 nm, with the associated theoretical wavelengths and corresponding mode orders shown in Table 1. The evolution of the chi squared parameter as a function of *g* is shown in Fig. 5 (b), demonstrating that the best solution is found near 3 nm. The effective material index in the presence of the gold thin film of 3.1 is clearly strongly modified from its standard value of 2.75, with this modification attributed to the formation of surface plasmon polariton waves at the gold film surface.

In the following argument, we affirm our assertion that SPPs are responsible for the effects observed: It has been shown in the literature^22^ that the prominence of light-matter interactions in a given spectral region depends on the difference between the optical frequency of the probing light and the characteristic frequency of the other phenomenon (eg. exciton generation or surface plasmon oscillation); the closer these frequencies are, the more prominent the coupling. The optical frequencies that we are interested in (corresponding to the free-space wavelengths present in the NBE spectrum of the nanoribbon centered on 680 nm) are very close to the range of SPP resonant frequencies that we can expect to observe, as given by the well-known expression linking the SPP resonant frequency ω_SPP_ to the plasma frequency ω_P_ of the bulk material ( = 9.05/h for gold^42^): ω_SPP_^2^ = ω_P_^2^/(ε_∞_ + ε_d_). In this expression, ε_∞_ represents the dielectric constant of the metal at infinite frequency ( = 10.3 for gold^42^) and ε_d_ the dielectric constant of the neighboring medium (assumed constant). If we insert as values for ε_d_ the two limiting dielectric constants for the extremes of our system, i.e. ε_d_ = ε_air_ = 1 for an infinite air gap (g = ∞), and ε_d_ = ε_material_ = 7.6 for the nanoribbon in contact with the gold (g = 0), this expression yields an equivalent free-space wavelength corresponding to the SPP resonance of between 460 nm (g = ∞) and 580 nm (g = 0). Therefore, the proximity of the near band edge PL emission of the nanoribbon to this wavelength range leads us to assume that SPPs will play a significant role in the light-matter interactions we observe and are therefore the dominant mechanism for the modified dispersion relation. Finally, we note that including the sole consideration of SPP effects in our model provides a good agreement with the experimental data without the need to involve additional phenomena.

**Figure 6:**
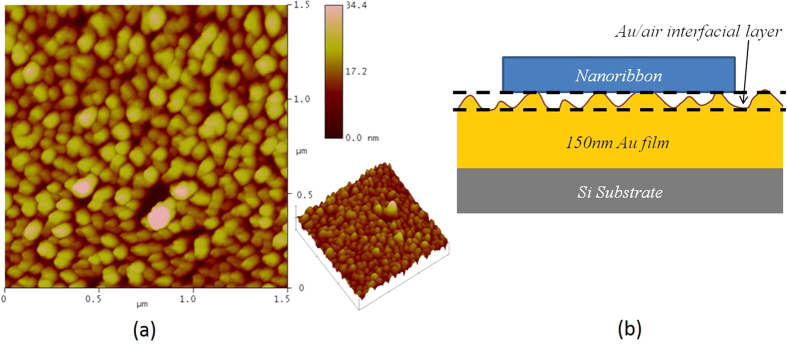
(**a**) 1.5*1.5 μm AFM surface scan of gold film surface, showing features with around 70 nm average lateral diameter and of the order of 1–10 nm height. (**b**) Schematic depiction of nanowire resting on rough gold surface with surface-averaged air gap between the two.

As mentioned earlier, the gold films upon which the NR has been placed for the purposes of this work is composed of grains with a mean in-plane diameter of 70 nm and an RMS roughness of ~3.5 nm as shown in Fig. 6 (a). We believe that the roughness inherent in these granular films limits the contact area with the NR, effectively providing a surface-averaged air gap and limiting undesirable charge transfer which could otherwise prevent the onset of amplified emission. This average air gap is represented by the gold-air interfacial layer beneath the nanoribbon depicted in Fig. 6 (b), and can be seen from Fig. 6 (a) to be of the order of 1–10 nm, therefore encompassing the value of 3.1 nm found by our model. Conversely, it can be seen that the surface roughness of the gold is sufficiently low so as not to prevent the modelling of the metal as a smooth layer in the eigenmode simulations from accurately representing the true substrate properties, nor does it prevent the formation and propagation of SPPs over the several tens of microns required for round-trips within the nanoribbon. The latter point is supported by the literature, where SPPs have been consistently demonstrated on thermally evaporated gold with surface features of the order of 1–100 nm in height and 10–1000 nm in lateral extent^34^,^35^,^43^. We conclude that the gold films prepared by magnetron sputtering used in this work provide a viable alternative for plasmonic applications where simpler architectures that avoid the need for a dielectric spacer, or inexpensive processing conditions are preferred to the more complex and demanding conditions required to form epitaxial gold films.

## Conclusion

In this work, we have demonstrated amplified spontaneous emission within the NBE photoluminescence spectrum of a dielectric nanostructure, that of a CdSSe nanoribbon. The association of this nanostructure with a metal coated substrate allows for the formation of a hybrid photonic-plasmonic cavity at the metal surface over the area delimited by the nanoribbon, leading to an increase in the wavenumber of light and an associated wavelength compression. The optical system described has been studied using an original modal analysis method in order to determine the effective index of modes in the plasmonic waveguide system, assuming an infinite extension in the longitudinal (*x*) direction. These effective modal indices have then been translated to the dielectric’s effective refractive index by taking into account the finite cavity length in order to successfully reproduce the resonant cavity wavelengths observed experimentally using a k-space analysis method.

## Additional Information

**How to cite this article**: Wood, T. *et al.* Resonance modulated amplified emission from CdSSe nanoribbons. *Sci. Rep.* **5**, 15071; doi: 10.1038/srep15071 (2015).

## Figures and Tables

**Table 1:** Observed resonant wavelengths from experimental photoluminescence (PL) measurements and analytic resolution results with (model 2) and without (model 1) SPP material effective index modification.

Experimentalmeasurements	Model 1: Photoniccavity prediction(unmodified materialindex, *n* = 2.75)		Model 2: Hybridcavity prediction(SPP modified materialindex, *N*_*material*_ = 3.10)	
Resonancewavelength(nm)	Resonancewavelength(nm)	Modeorder(*l,m*)	Resonancewavelength(nm)	Modeorder(*l,m*)
684	684.1	31,8	683.5	47,8
689	688.7	30,8	689.0	46,8
694	693.3	29,8	694.6	45,8
700	697.8	28,8	700.2	44,8
706	702.2	27,8	705.7	43,8
*χ*^2^*(λ*_*exp*_*,λ*_*theory*_)	*20.236*		*0.701*	
